# Repositioning Drugs for Psychiatry

**Published:** 2020

**Authors:** Shahin Akhondzadeh

**Affiliations:** Psychiatric Research Center, Roozbeh Hospital, Tehran University of Medical Sciences, Tehran, Iran

Drug development can be time-consuming and expensive. Recent estimates suggest that, on average, it takes 10 years and at least $1 billion to bring a drug to market. Since last decade, 30–40% of drugs or biologics that were approved or launched for the first time in the US were either drugs repositioned for new indications, reformulations or new combinations of existing drugs. This is the lifecycle business with repositioning as a major contributor, and it is rarely given much attention outside of its practitioners 1,2.

In general, drug repurposing or drug repositioning alludes to the development of existing drugs or pro-drugs for new indications, not necessarily related to the original disease focus. These drugs have probably failed in late-stage clinical trials by lacking in efficacy or safety, or have problems associated with commercial strategies, patent expiration or geographic expansion. Repositioning existing drug substances for the treatment of different indications can significantly reduce the cost and time required for the development of new medicines. Therefore, drug repurposing brings forth the benefit of quickening patient access to innovative and effective treatment at lower risk and development cost for the industry 1,2. There are a number of different definitions of drug repurposing. All of them contain two key elements:

Taking existing scientific or medical knowledge and technology that is “approved” for human use in one disease or condition; and

Applying this knowledge and technology to another disease or condition.

Aspirin, a drug that’s been in use in some form or other for many hundreds of years was originally employed, and indeed still is, as a mild pain-relieving analgesic. But it’s probably more commonly used today as an antiplatelet agent helping to prevent blood clotting that can occur in thromboembolic disease.

Nervous system diseases represent a major health concern worldwide. Although important financial and professional investment, their etiology and pathophysiology still remain mostly elusive. Moreover, the clinical need of disease-modifying therapies is still unmet. In medicine in general and in psychiatry in particular, repositioning was the result of serendipitous but astute clinical observation of an unexpected benefit or expected or unexpected adverse effects 3. A number of old drugs have been reintroduced for psychiatric indications such as celecoxib for schizophrenia, tamoxifen for mania and scopolamine for depression 4–7. Drug repurposing has become a new business segment for the life science services industry. In conclusion, drug repurposing emerges as a new value proposition for the industry, patients and payers.
